# Accuracy of non-invasive sensors measuring core body temperature in cardiac surgery ICU patients – results from a monocentric prospective observational study

**DOI:** 10.1007/s10877-023-01049-7

**Published:** 2023-07-12

**Authors:** Georg Engelbart, Sebastian Brandt, Tobias Scheeren, Alexander Tzabazis, Oliver Kimberger, Patrick Kellner

**Affiliations:** 1 Department of Anaesthesiology and Intensive Care Medicine, University Hospital Schleswig-Holstein, Ratzeburger Allee 160, D-23538 Lübeck, Germany; 2 Department of Anesthesiology and Intensive Care Medicine, Städtisches Klinikum Dessau, Brandenburg Medical School Theodore Fontane, Dessau, Germany; 3 Department of Anaesthesia, Intensive Care Medicine and Pain Medicine, Medical University of Vienna, Vienna, Austria; 4 Ludwig Boltzmann Institute for Digital Health and Patient Safety, Medical University of Vienna, Vienna, Austria

**Keywords:** Zero-heat-flux, Double-sensor, Temperature monitoring, Perioperative hypothermia, Core temperature, Non-invasive monitoring

## Abstract

**Purpose:**

Temperature monitoring in the perioperative setting often represents a compromise between accuracy, invasiveness of probe placement, and patient comfort. Transcutaneous sensors using the Zero-Heat-Flux (ZHF) and Double-Sensor (DS) technology have been developed and evaluated in a variety of clinical settings. The present study is the first to compare the performance of both sensors simultaneously with temperature measured by a Swan-Ganz catheter (PAC) in patients admitted to the intensive care unit (ICU) after cardiac surgery.

**Methods:**

In this monocentric prospective observational study patients were postoperatively transferred to the ICU and both sensors were placed on the patients’ foreheads. Core body temperature measured by intraoperatively placed PAC served as gold standard. Measurements were recorded at 5-minute intervals and up to 40 data sets per patient were recorded. Bland and Altman’s method for repeated measurements was used to analyse agreement. Subgroup analyses for gender, body-mass-index, core temperature, airway status and different time intervals were performed. Lin’s concordance correlation coefficient (LCCC) was calculated, as well as sensitivity and specificity for detecting hyperthermia (≥ 38 °C) and hypothermia (< 36 °C).

**Results:**

Over a period of six month, we collected 1600 sets of DS, ZHF, and PAC measurements, from a total of 40 patients. Bland-Altman analysis revealed a mean bias of -0.82 ± 1.27 °C (average ± 95% Limits-of-Agreement (LoA)) and − 0.54 ± 1.14 °C for DS and ZHF, respectively. The LCCC was 0.5 (DS) and 0.63 (ZHF). Mean bias was significantly higher in hyperthermic and hypothermic patients. Sensitivity and specificity were 0.12 / 0.99 (DS) and 0.35 / 1.0 (ZHF) for hyperthermia and 0.95 / 0.72 (DS) and 1.0 / 0.85 (ZHF) for hypothermia.

**Conclusion:**

Core temperature was generally underestimated by the non-invasive approaches. In our study, ZHF outperformed DS. In terms of agreement, results for both sensors were outside the range that is considered clinically acceptable. Nevertheless, both sensors might be adequate to detect postoperative hypothermia reliably when more invasive methods are not available or appropriate.

**Trial Registration:**

German Register of Clinical Trials (DRKS-ID: DRKS00027003), retrospectively registered 10/28/2021.

**Supplementary Information:**

The online version contains supplementary material available at 10.1007/s10877-023-01049-7.

## Introduction

Unintentional perioperative hypothermia is a challenge for anaesthesiologists in all surgical specialties. Patients under general [1] and regional anaesthesia [2] experience interference with their physiological thermoregulatory system, such as heat redistribution from the core to the periphery. The incidence of perioperative hypothermia has been reported to be as high as 20 to 70% [3, 4] Negative effects, e.g. altered pharmacodynamics, increase in surgical site infections, and coagulopathy have been described and are well accepted [5]. Despite recommendations by national [6] and international guidelines [7] for monitoring core body temperature as well as prevention and treatment of perioperative hypothermia the clinical implementation is still incomplete [8]. Requirements for ideal temperature monitoring are (1) non-invasiveness, (2) high accuracy, and (3) continuous monitoring. Clinical standard are usually invasive monitoring sites, such as nasopharyngeal, oesophageal, vesical, and rectal [9]. Probe placement has, however, a potential for complications, e.g. nosebleed, and substantial impact on patients’ comfort in the recovery room and ICU. For patients undergoing regional anaesthesia and minor procedures under general anaesthesia less invasive techniques often lack measurement accuracy in contrast to the gold standard, defined as direct measurement in the pulmonary artery [9–11]. Recent technologies such as the transcutaneous zero-heat-flux (ZHF), e.g. Bair Hugger™ (3 M, Saint Paul, Minnesota, USA) or double-sensor (DS) technique, e.g. Tcore™ (Drägerwerk AG & Co. KGaA, Lübeck, Germany) are using disposable surface sensors placed on the patient’s forehead. While these technologies benefit patient comfort, there is insufficient data available for accuracy of temperature measurements.

While several studies reported promising results for individual sensors, there is limited data available investigating both sensors in direct comparison to the gold standard [12]. To our knowledge, our study is the first to simultaneously investigate the accuracy of both non-invasive sensors compared to the gold standard in an ICU setting in cardiac surgery patients.

## Methods

### Study design and approval

After approval of the ethics committee of the University of Lübeck (Reference: 20–090 of April 9th, 2020), we conducted a monocentric prospective observational study at the Department of Anaesthesiology and Intensive Care Medicine at the University Medical Center Schleswig-Holstein, Lübeck Campus, which was carried out from 10/2020 to 04/2021. The study was designed and performed in accordance with the guidelines of the Declaration of Helsinki [13] and the requirements of the European Union’s General Data Protection Regulation [14]. Written informed consent was obtained from all participants before inclusion in the study. The trial was retrospectively registered with the German Register of Clinical Trials (DRKS-ID: DRKS00027003).

### Study population

Inclusion criteria were: 18 years or older, capacity to give informed consent, and clinical indication for placement of a pulmonary artery catheter (PAC). All participants were planned for elective cardiac surgery at the Department of Cardiac and Thoracic Vascular Surgery at the University Medical Center Schleswig-Holstein. The clinical decision for placement of the PAC was ultimately made by the attending anaesthesiologists, who were not directly involved in this study. Patients, who needed emergency surgery, were excluded.

### Study procedures

After implementation of monitoring and induction of general anaesthesia according to hospital standards, a venous sheath (Arrow-Flex® Sheath: 9 Fr., Model SI-09903-E, Teleflex Medical Europe Ltd., Westmeath, Ireland) was placed, usually in the right internal jugular vein. A PAC (Swan-Ganz Advanced Technology Catheter, CCOmbo RVEDV, Model 774F75, Fa. Edwards Lifesciences Corporation, Irvine, California, USA) was inserted via the sheath for perioperative hemodynamic monitoring before skin incision. After completion of the surgical procedure and transfer to the ICU, both non-invasive sensors (DS and ZHF) were placed on the patient’s forehead according to manufacturers’ guidelines, opposite to each other. PAC-data was recorded via HemoSphere™ Monitoring System (Edwards Lifesciences Corporation, Irvine, California, USA). After preparation of the patient, the ZHF and DS were simultaneously connected to a separate study monitor (Infinity® Delta Monitor, Drägerwerk AG & Co. KGaA, Lübeck, Germany). Data collection began when measurements of both sensors became available, using DataGrabber software (Version 0.08, Drägerwerk AG & Co. KGaA, Lübeck, Germany). Temperature measurements were recorded as T_DS_ (Double-Sensor temperature), T_ZHF_ (Zero-Heat-Flux temperature), and T_PAC_ (Pulmonal artery temperature), respectively. Measurements were taken every 5 min for at least 3 h and 15 min yielding 40 data pairs for each sensor per patient.

### Statistical methods

We assessed agreement between both non-invasive and PAC temperature readings using Bland Altman-analysis for repeated measurements, calculating the mean bias and 95%-limits-of-agreement (LoA: mean bias ± 1.96 × standard deviation) for each sensor, respectively [15, 16]. According to comparable studies, clinically acceptable deviation was defined as having 95% of differences within ± 0.5 °C compared to the gold standard [12]. The difference in mean bias between non-invasive sensors was calculated and checked for significance using a two-tailed paired t-test. Additionally, we calculated Lin’s concordance correlation coefficient (LCCC) to evaluate the strength of agreement in continuous measurements [17]. Subgroup analyses were performed to investigate effects of gender, body-mass-index (> 30 vs. = < 30), airway (non-intubated vs. intubated), and temperature (hyper-, normo- or hypothermia defined as ≥ 38 °C, 36–37,9 °C and < 36 °C, respectively). Mean bias was reviewed for each time point to detect changes in accuracy during the course of measurement.

Sensitivity and specificity for the detection of hyperthermia and hypothermia were calculated. In addition to this, receiver-operating-characteristic (ROC) curves were generated to compare the diagnostic performance of DS and ZHF. Area-under-the-curve (AUC) and Youden index [18, 19] were also calculated.

Raw data were processed, and tables were created using Microsoft Excel® (Version 2019, Microsoft, Redmond, Washington, USA). Statistical and graphical analyses were performed using MedCalc® (Version 20.015, MedCalc Software Ltd, Ostend, Belgium). Figures were created or edited using Microsoft Paint® (version Windows® 10) and Microsoft PowerPoint® (Version 2019, both Microsoft, Redmond, Washington, USA).

Results were considered significant for p < 0.05.

### Sample size calculation

Sample size calculation was performed based on the publications of Kimberger et al. [20] and Eshraghi et al. [21] and an expected mean bias of -0.07 °C and standard deviation of 0.2 °C using MedCalc® Sample Size Calculator (MedCalc Software Ltd, Ostend, Belgium) for Bland-Altman analysis (BA-analysis). This resulted in the need to assess n = 629 data pairs. We increased to n = 1600, split into 40 data pairs per patient and a total of 40 patients.

## Results

**Fig. 1:**
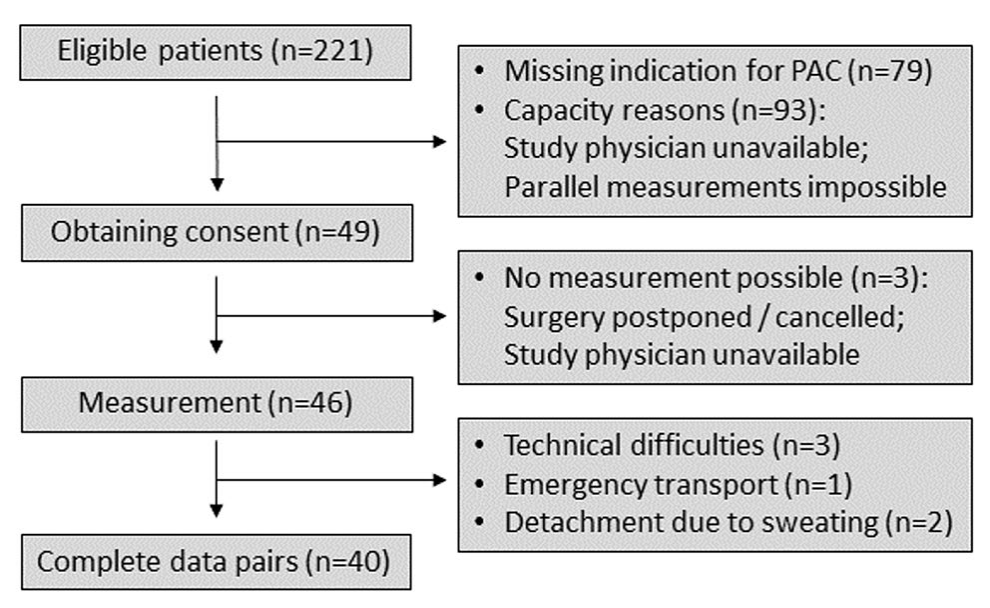
Patient flow in accordance with CONSORT [22]

From November 2020 to April 2021, 221 eligible patients were identified. After exclusion of 181 patients, forty patients completed the study and data were analysed. A detailed patient flow according to CONSORT is shown in Fig. 1.

### Patient characteristics and data distribution

Epidemiologic data, surgical procedure, and airway management in ICU are presented in Table 1.

Based on data structure and by drawing histograms, we assumed a sufficient normal distribution of the differences (T_DS_-T_PAK_ and T_ZHF_-T_PAK_) as needed for the Bland-Altman analysis [16]. We performed an outlier analysis according to Tukey [23] which classified 32 differences as outliers (zero far-out values) for the DS and 84 outliers (24 far-out values) for the ZHF, respectively. More detailed information on data distribution outliers and excluded patients is provided in Online Resource 1–4.

**Table 1:** Characteristics of study population

	Mean (SD)	Range
**Age [years]**	68.3 (8.6)	52–83
**Temperature [°C]**	37.2 (0.8)	34.6–39.0
**BMI** ^**a**^ **[kg/m²]**	28.7 (5.7)	20.7–45.0
	**Total**	**Fraction [%]**
**All patients**	40	100
**Gender**		
Male	26	65
„Female	14	35
**BMI** ^**a**^ **[kg/m²]**		
BMI ≥ 30	12	30
BMI < 30	28	70
**Airway in ICU**		
Extubated^b^	30	75
Intubated	10	25
**Temperature**		
Ever hyperthermic	13	32.5
Ever hypothermic	10	25
**Operation**		
OPCABG^c^	20	50
CABG^d^	2	5
MICS-AVR^e^	4	10
AVR + CABG	5	12.5
MICS-MVR^f^	3	7.5
Other	6	15

^a^Body mass index; ^b^Extubation during study period in ICU

^c^Off-pump coronary artery bypass graft;

^d^Coronary artery bypass graft;

^e^Minimally invasive cardiac surgery – aortic valve replacement;

^f^Minimally invasive cardiac surgery – mitral valve repair

### Bland-Altman analysis

We included the differences of 1600 data pairs per sensor in the BA-analysis. Mean bias, 95% LoA, proportion of differences within ± 0.5 °C and LCCC are given in Table 2.

**Table 2:** Results of Bland-Altman analysis

	Mean bias (SD) [°C]	Lower 95% LoA^a^ (CI) [°C]	Upper 95% LoA^a^ (CI) [°C]	Proportion within ± 0.5 °C	LCCC^b^ (CI)
**All data pairs (n = 1600)**					
Double-Sensor	-0.82 (0.64)	-2.09 (-2.44; -1.84)	0.45 (0.19; 0.79)	0.36	0.5 (0.47; 0.52)
Zero-Heat-Flux	-0.54 (0.58)	-1.68 (-1.97; -1.46)	0.6 (0.39; 0.89)	0.61	0.63 (0.61; 0.66)

^a^Limits-of-agreement; ^b^Lin’s concordance correlation coefficient

The difference between the mean bias of DS and ZHF was 0.28 °C (CI: 0.24; 0.32 °C) and proofed significant by t-test (p = < 0.0001). Based on the suggested grading method by Mc Bride et al. [24], the LCCC for both sensors showed a weak agreement with PAC measurements.

**Fig. 2:**
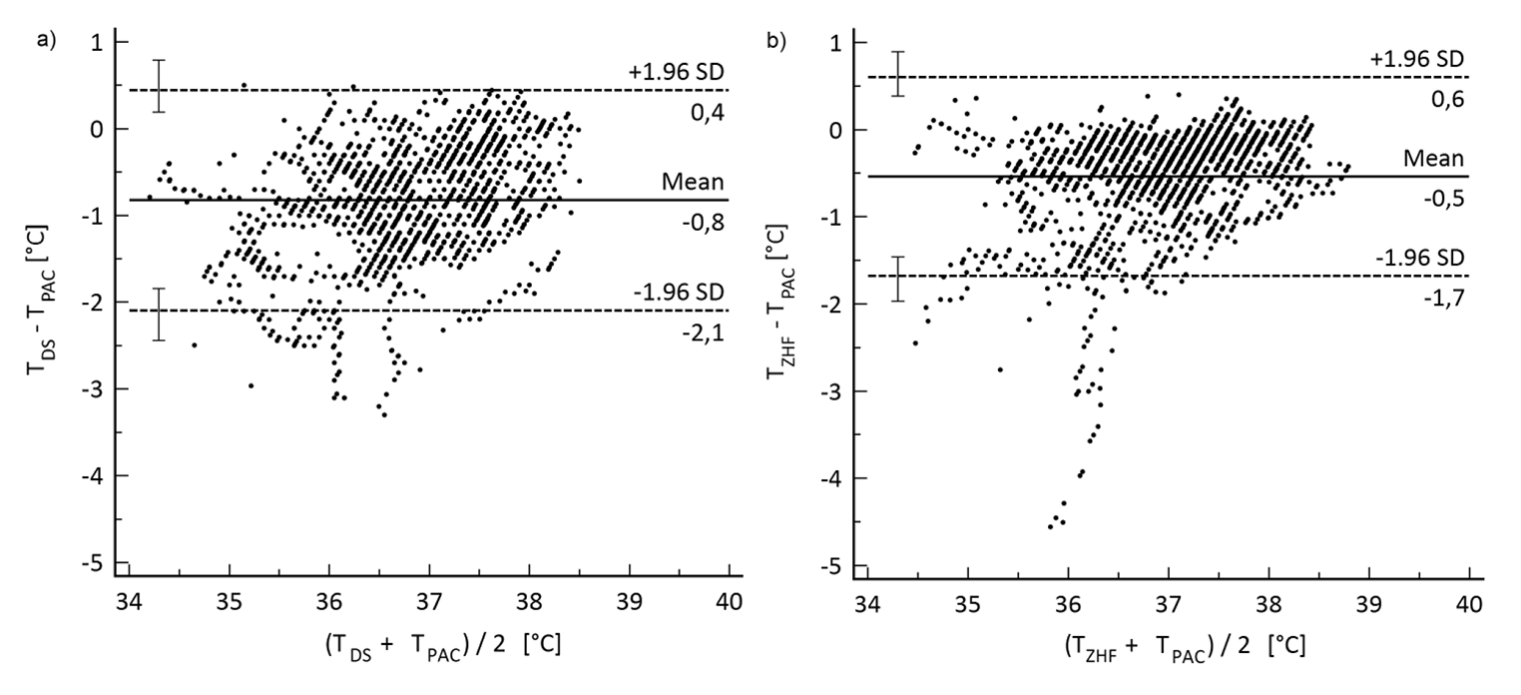
Bland-Altman plots for (**a**) Double-Sensor and (**b**) Zero-Heat-Flux-Sensor T_DS_: Double-Sensor temperature; T_ZHF_: Zero-Heat-Flux temperature; T_PAC_: Pulmonal artery temperature

The differences of the non-invasive measurements compared with the PAC including outliers are plotted in Fig. 2 using a BA-diagram. The percentage of negative differences was 90.9% (DS, Fig. 2a) and 93.2% (ZHF, Fig. 2b), respectively.

Subsequently, a separate analysis of subgroups was performed. Results are shown Online Resource 5. Significant differences for the DS were found for hyperthermic and hypothermic measurements (difference of mean bias − 0.14 °C and − 0.13 °C, respectively, compared to the normothermic group; p = 0.002 and p = 0.0424, respectively). For ZHF, this difference was found only in the hyperthermic group (-0.17 °C; p = < 0.0001). Difference between the male and female groups was − 0.44 °C for DS and − 0.14 °C for ZHF, respectively (p = < 0.0001).

To estimate agreement of the methods over the course of measurement, mean bias for each timepoint was formed. Steady temperature readings were achieved after 10 min. Interestingly, mean bias for DS remained relatively stable, while ZHF readings began to decrease after 1,5 h, resulting in increasing mean bias of up to -0,69 °C. Detailed results are shown in Online Resource 6.

### Detection of hyper- / hypothermia

Sensitivity and specificity for detection of hyper- / hypothermia were calculated for commonly used thresholds of ≥ 38,0 °C and < 36,0 °C (see Table 3) and corresponding ROC-curves for detection of hyper- and hypothermia are shown in Online Resource 7.

**Table 3:** Sensitivity and specificity for hyper/hypothermia

	Threshold^b^	Sensitivity	Specificity
**Hyperthermia** ^**a**^			
Double-Sensor	≥ 38.0 °C	0.12	0.99
Zero-Heat-Flux	≥ 38.0 °C	0.35	1
**Hypothermia** ^**a**^			
Double-Sensor	< 36.0 °C	0.95	0.72
Zero-Heat-Flux	< 36.0 °C	1	0.85

^a^Defined by corresponding PAC temperature reading ≥ 38.0 °C or < 36.0 °C, respectively

^b^Referring to the non-invasive temperature readings

To compare the two sensors in terms of their test performance the AUC was calculated. A significant difference in AUC in favour of the ZHF was found only for hyperthermia (difference 0.05; p = 0.0099) but not for hypothermia (difference 0.017; p = 0.1115). Detailed results of the AUC-ROC analysis can be found in Table 4.

**Table 4:** Results of AUC-ROC analysis for hyper- / hypothermia

	AUC (CI)	Youden Index	Associated threshold^b^	Sensitivity	Specificity
**ROC curve - hyperthermia** ^**a**^					
Double-Sensor	0.84 (0.82; 0.85)	0.58	> 36.9 °C	0.85	0.73
Zero-Heat-Flux	0.89 (0.87; 0.9)	0.61	> 37.4 °C	0.73	0.88
**ROC curve - hypothermia** ^**a**^					
Double-Sensor	0.93 (0.92; 0.94)	0.74	≤ 35.8 °C	0.92	0.82
Zero-Heat-Flux	0.95 (0.94; 0.96)	0.85	≤ 36.0 °C	1	0.85

^a^Defined by corresponding PAC temperature reading ≥ 38.0 °C or < 36.0 °C, respectively

^b^Referring to the non-invasive temperature readings

## Discussion

Perioperative temperature management and continuous core temperature monitoring remain challenging for both intensivists and anaesthesiologists. Even when invasive methods are appropriate, there are potential disadvantages. For example, nasopharyngeal probes can cause severe epistaxis in anticoagulated patients [25] and the accuracy of urinary bladder probes depends on diuresis [26]. Less-invasive technologies have been introduced but compromise accuracy. Up to now there is only limited evidence for reliability of results obtained using DS and ZHF technologies, especially in ICU patients. Results from our study show non-invasive sensors did not meet the target criteria, i.e. 95% of differences within the ± 0.5 °C range when compared to PAC readings. Although we found a significant advantage of the ZHF over the DS, both technologies show a general tendency to underestimate core temperature with high mean bias, wide LoA and weak agreement in the LCCC. Our results indicate that differences in measurement accuracy is partially explained by interindividual variations. While there was excellent agreement between methods in a subset of patients, correlation and accuracy were consistently poor in other patients. Details can be found at Online Resource 8. These might be an interesting finding, but unfortunately, the subgroup analysis couldn’t achieve further clarification which patients can be temperature monitored using a less-invasive approach with reasonably good accuracy. Although we saw some divergence in mean bias and LoA for hyper- and hypothermic values, results mainly indicate that the agreement tends to decrease even more over time.

In a comparable ICU patient population Dahyot-Fizeler et al. [27] and Braeuer et al. [28] reported a mean bias of 0.0 and − 0.12 °C, respectively, with narrower LoA for the ZHF. While Dahyot-Fizeler studied a population of only 7 patients after cardiac surgery, using femoral arterial temperature, i.e. not the gold standard, as reference, Braeuer investigated 50 patients in a mixed ICU population, comparing the ZHF to iliac and pulmonary measurements. Eshraghi et al. [21] were the first to investigate the agreement of ZHF and PAC measurements in 2014 and found a mean bias of -0.23 °C with LoA of -1.05 to 0.59 °C in a group of 105 patients measured both intra- and postoperatively in the ICU. Conducting their study in an intraoperative setting Verheyden et al. [29], Boisson et al. [30] and Maekinen et al. [31] found mean biases of -0.06, -0.06 and − 0.05 °C with LoA of -0.95 to 0.83, -0.41 to 0.29 and − 0.56 to 0.47 °C, respectively. Included patients underwent mostly cardio-vascular surgery except for Boisson’s study who investigated a population undergoing abdominal surgery.

For the DS, available data are more limited. While Soehle et al. [32] found excellent agreement with a mean bias of -0.02 °C and narrow LoA in their study on 22 patients undergoing intraabdominal surgery, the results of Sastre et al. [33] in a cardiac surgical population of 40 patients are more comparable to our findings. Reported LoA range from − 1.36 to 0.96 with a mean bias of -0.2 °C, and only 55% of differences within the ± 0.5 °C range. All publications mentioned above support our conclusion that there is a tendency for the non-invasive sensors to underestimate core temperature. Whether this underestimation is clinically significant and potentially harmful, needs to be determined. A recent meta-analysis of Conway et al. [34] pooled data from 16 different publications investigating ZHF in comparison to a variety of reference methods. Although the authors found a mean bias of 0.03 °C, i.e. no general underestimation of core temperature, the LoA range was − 0,93 to 0,98 °C, exceeding the predefined limits of ± 0.5 °C.

Janke et al. [35] used an oesophageal probe in 25 patients, demonstrating similarly wide LoA of -1.02 and 1.07 °C.

Studies that compare DS and ZHF to each other are very limited. Apart from our study, only Gomez-Romero et al. [36] investigated the performance of DS and ZHF simultaneously in a population of 41 patients during elective valvular heart surgery. Similar to our findings, their results favoured the ZHF over DS. Compared with PAC temperature mean bias was − 0,21 and − 0,48 °C, respectively, with LoA exceeding ± 2 °C, i.e. much wider compared to our findings.

Most authors do not provide data or results of individual participants, but Janke et al. [35] report that 6 out of 25 patients exhibited a substantial difference, i.e. mean bias, compared to the remaining 19 patients. In those cases, bias increased over the course of measurement, which was also observed in our study. Since their subgroup results showed no influence of body-mass-index, ethnicity, gender, and dosage of vasoactive drugs, the reason for this difference remains unclear. Sensor detachment over time could be a potential reason for these findings but was not formally assessed in our and Janke’s study.

Regarding the detection of hypo- and hyperthermia, only Braeuer et al. [28] and Gómez-Romero et al. [36] determined the sensitivity and specificity of the investigated sensors and reported findings in line with our results. Based on this data, both the ZHF and the DS seem suitable for diagnosing (intraoperative) hypothermia. However, for the detection of hyperthermia and fever with potential therapeutic consequences, both sensors’ sensitivity seems to be too low. In addition, there is a bias towards underestimating temperature. A significant difference between the two sensors when comparing AUCs in ROC analysis could only be determined for hyperthermia.

### Limitations

There are some limitations that might explain why the overall agreement in our study is worse compared to some of the previously reported research. Most of the available studies have been conducted in an intraoperative setting, making it easier to closely monitor proper adhesion of the sensor to the patients’ skin. During surgery, there is usually less patient movement and manipulation in general, compared to our patient population undergoing standard treatment in the ICU such as oral care.

In contrast to many of the studies summarized above, no additional equilibration time was granted before starting data recording. Some authors describe up to 15 min of delay between sensor placement and first temperature recording.

A potential confounder could be adhesion quality of the non-invasive sensors, which could be impacted by sweating. While we did not formally assess extent of sweating, two patients had to be excluded for this reason. We also did not document administration of vasopressors that might affect skin perfusion and hence measurement quality.

A relatively high number of outliers, especially for ZHF, may also have contributed to the outcome of this study. We did not exclude those values from our analysis. The majority of outliers were obtained from two patients, see Online Resource 3 and 4 for details. The effect on the results of our study could possibly have been reduced by a larger sample size in the sense of a higher number of patients. However, related studies included a similar or smaller number of participants.

## Conclusion

Based on our results obtained from a patient population in the intensive care unit after cardiac surgery, both the DS and the ZHF showed limited agreement with core body temperature determined by invasive blood temperature using Swan-Ganz Catheter. Comparing the performance of both methods, our results suggests that there might be advantage for the ZHF in terms of agreement. Further studies and robust meta-analyses should be performed especially regarding the DS. Important confounders contributing to measurement inaccuracy in some individuals have yet to be identified.

Regarding the detection of hypothermia, the high sensitivity and sufficient specificity obtained in this study agree with data from other authors and indicate that DS and ZHF could be a feasible solution for perioperative detection of hypothermia.

Taken together Double-Sensor as well as Heat-Flux-Sensor have lacs of agreement compared with an invasive measurement of temperature using Swan-Ganz Catheter, but especially in detection of hypothermia they could be an option in ICU.

### Electronic supplementary material

Supplementary material
