# Supplementary material for: Accuracy of non-invasive sensors measuring core body temperature in cardiac surgery ICU patients – results from a monocentric prospective observational study
Source: J Clin Monit Comput. 2023 Jul 12;37(6):1619–26. doi: 10.1007/s10877-023-01049-7 (PMC10651547; doi:10.1007/s10877-023-01049-7)
Supplement: Supplementary file 1 — Supplementary material [file 10877_2023_1049_MOESM1_ESM.doc]

# Online Resource / Supplement

**Online Resource 1** Characteristics of secondary excluded patients

| **ID^a^** | **Age [years]** | **Sex** | **BMI^b^ [kg/m²]** | **Operation** | **Mean Temp (SD) [°C]** | **Temp Range**  **[°C]** | **Complete data pairs** | **Problem** |
| --- | --- | --- | --- | --- | --- | --- | --- | --- |
| **1** | 60 | m | 27.8 | N/A | / | / | 0 | Technical^f^ |
| **3** | 54 | m | 26.5 | N/A | 37.7 (0.2) | 37.4-38.1 | 19 | Technical^f^ |
| **36** | 57 | m | 34.9 | MIC-MKR^c^ | 38.6 (0.1) | 38.3-38.7 | 6 | Sweating^g^ |
| **38** | 66 | m | 36.7 | OPCAB^d^ | 38.5 (0.3) | 38.0-38.9 | 33 | Sweating^g^ |
| **41** | 69 | m | 26.9 | AKE + Asc.^e^ | 38.1 (0.1) | 37.9-38.1 | 20 | Transport^h^ |
| **45** | 61 | m | 27 | OPCAB^c^ | 37.5 (0.2) | 37.7-37.9 | 13 | Technical^f^ |

^a^Patient ID; ^b^Body mass index; ^c^Minimally invasive cardiac surgery - mitral valve repair; ^d^Off-pump coronary artery bypass graft; ^e^Aortic valve replacement and replacement of the ascending aorta; ^f^Technical errors, including configuration error of patient monitor and accidental disconnection of power source for study equipment; ^g^Detachment of Zero-Heat-Flux-Sensor due to excessive sweating; ^h^Discontinuation of measurements due to emergency CT-scan

**Online Resource 2** Distribution plot and outliers for a) Double-Sensor and b) Zero-Heat-Flux-Sensor


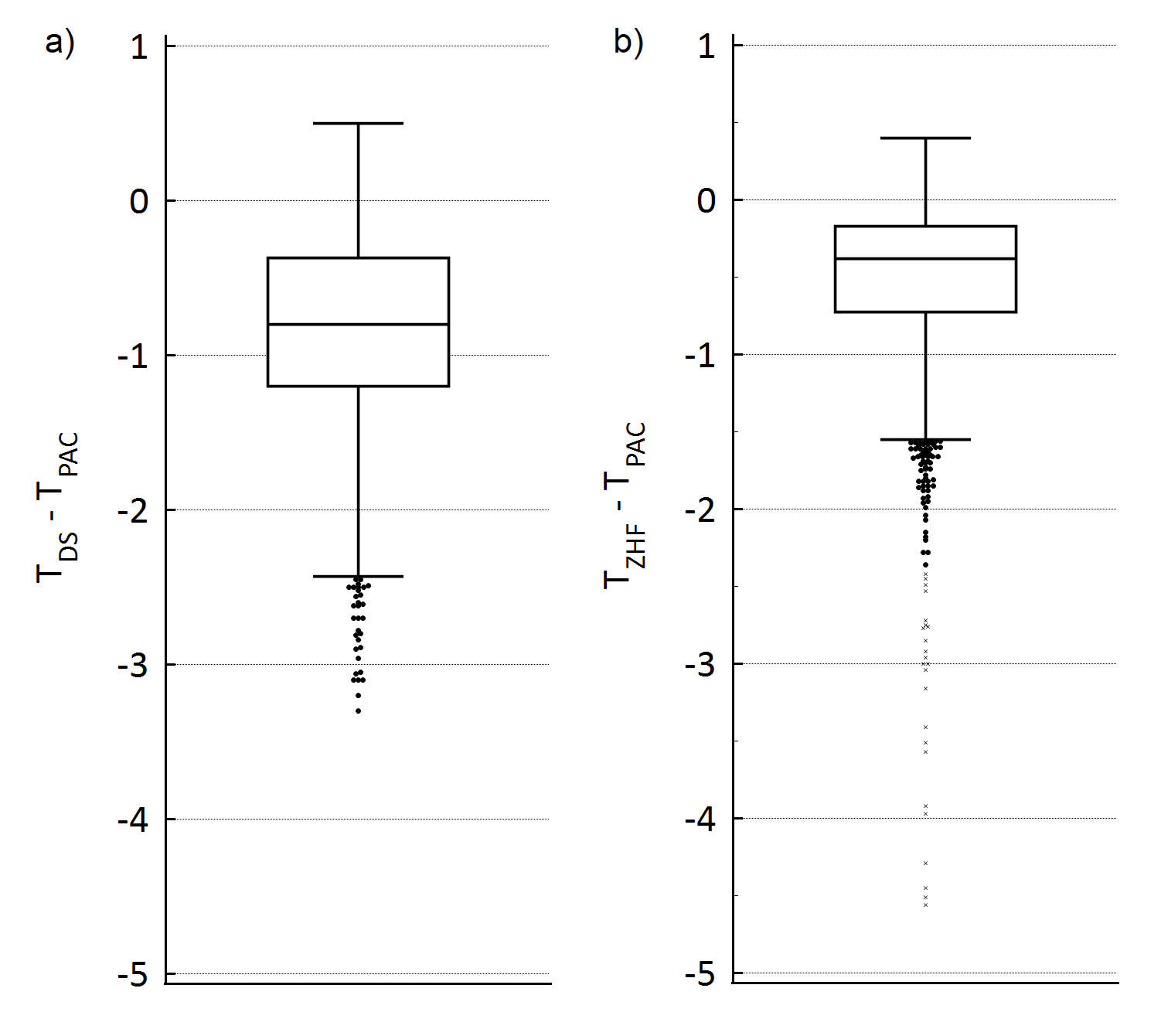


T_DS_: Double-Sensor temperature; T_ZHF_: Zero-Heat-Flux temperature; T_PAC_: Pulmonal artery temperature

**Online Resource 3** Analysis of outliers for Double-Sensor data

|  | **Outside values^a^** | **Far-out values^a^** |
| --- | --- | --- |
| **Range [°C]** | -2.45 to -3.3 | N/A |
|  |  |  |
| **Total** | 32 | 0 |
| **Count per Pat.-ID** | | |
| ID 7 | 1 | 0 |
| ID 23 | 5 | 0 |
| ID 25 | 1 | 0 |
| ID 34 | 10 | 0 |
| ID 40 | 1 | 0 |
| ID 44 | 14 | 0 |

^a^According to Tukey et al. [Tukey, J. W. (1977). Exploratory data analysis. Reading, Mass: Addison-Wesley Pub. Co.]

**Online Resource 4** Analysis of outliers for Zero-Heat-Flux data

|  | **Outside values^a^** | **Far-out values^a^** |
| --- | --- | --- |
| **Range [°C]** | -1.56 to -2.36 | -2.42 to -4.56 |
|  |  |  |
| **Total** | 60 | 24 |
| **Count per Pat.-ID** | | |
| ID 4 | 1 | 0 |
| ID 10 | 3 | 0 |
| ID 16 | 5 | 0 |
| ID 17 | 23 | 1 |
| ID 19 | 2 | 0 |
| ID 25 | 1 | 0 |
| ID 34 | 12 | 13 |
| ID 44 | 13 | 10 |

^a^According to Tukey et al. [Tukey, J. W. (1977). Exploratory data analysis. Reading, Mass: Addison-Wesley Pub. Co.]

**Online Resource 5** Complete results of subgroup analysis

| **Subset** | **Mean bias (SD)**  **[°C]** | **Lower 95% LoA^a^ (CI)  [°C]** | **Upper 95% LoA^a^ (CI)  [°C]** | **Proportion**  **within ± 0.5 °C** | **LCCC^b^ (CI)** |
| --- | --- | --- | --- | --- | --- |
| **Hyperthermia (n=241)** | | | | | |
| Double-Sensor | -0.96 (0.65) | -2.51 (-3.37; -2.04) | 0.6 (0.13; 1.45) | 0.23 | 0.05 (0.02; 0.08) |
| Zero-Heat-Flux | -0.72 (0.78) | -2.82 (-4.25; -2.02) | 1.39 (0.59; 2.82) | 0.53 | 0.14 (0.1; 0.18) |
| **Normothermia (n=1250)** | | | | | |
| Double-Sensor | -0.82 (0.64) | -2.08 (-2.46; -1.81) | 0.45 (0.17; 0.82) | 0.39 | 0.34 (0.31; 0.37) |
| Zero-Heat-Flux | -0.55 (0.52) | -1.6 (-1.88; -1.38) | 0.5 (0.28; 0.78) | 0.62 | 0.48 (0.45; 0.51) |
| **Hypothermia (n=109)** | | | | | |
| Double-Sensor | -0.95 (0.65) | -2.23 (-3.3; -1.69) | 0.32 (-0.21; 1.39) | 0.65 | 0.17 (0.09; 0.24) |
| Zero-Heat-Flux | -0.6 (0.63) | -1.84 (-2.89; -1.33) | 0.65 (0.13; 1.69) | 0.52 | 0.16 (0.05; 0.27) |
| **Extubated (n=1200)** | | | | | |
| Double-Sensor | -0.82 (0.6) | -2.0 (-2.4; -1.73) | 0.38 (0.11; 0.77) | 0.35 | 0.55 (0.52; 0.57) |
| Zero-Heat-Flux | -0.54 (0.57) | -1.66 (-2.0; -1.41) | 0.58 (0.34; 0.93) | 0.61 | 0.66 (0.63; 0.69) |
| **Intubated (n=400)** | | | | | |
| Double-Sensor | -0.86 (0.75) | -2.3 (-3.55; -1.83) | 0.68 (0.11; 1.83) | 0.4 | 0.33 (0.28; 0.38) |
| Zero-Heat-Flux | -0.53 (0.61) | -1.77 (-2.68; -1.31) | 0.71 (0.25; 1.62) | 0.63 | 0.52 (0.46; 0.57) |
| **BMI^c^ < 30 kg/m² (n=1120)** | | | | | |
| Double-Sensor | -0.83 (0.63) | -2.09 (-2.52; -1.8) | 0.43 (0.14; 0.86) | 0.35 | 0.52 (0.49; 0.55) |
| Zero-Heat-Flux | -0.52 (0.57) | -1.65 (-2.01; -1.40) | 0.61 (0.36; 0.97) | 0.61 | 0.67 (0.64; 0.7) |
| **BMI^c^ ≥ 30 kg/m² (n=480)** | | | | | |
| Double-Sensor | -0.82 (0.66) | -2.16 (-3.02; -1.69) | 0.52 (0.06; 1.39) | 0.39 | 0.37 (0.33; 0.42) |
| Zero-Heat-Flux | -0.58 (0.57) | -1.77 (-2.53; -1.37) | 0.62 (0.21; 1.38) | 0.61 | 0.47 (0.42; 0.52) |
| **Male (n=1040)** | | | | | |
| Double-Sensor | -0.67 (0.51) | -1.68 (-2.02; -1.44) | 0.34 (0.11; 0.68) | 0.41 | 0.6 (0.58; 0.63) |
| Zero-Heat-Flux | -0.49 (0.45) | -1.38 (-1.69; -1.18) | 0.41 (0.21; 0.71) | 0.62 | 0.71 (0.68; 0.73) |
| **Female (n=560)** | | | | | |
| Double-Sensor | -1.11 (076) | -2.64 (-3.5; -1.5) | 0.41 (-0.08; 1.27) | 0.26 | 0.37 (0.32; 0.41) |
| Zero-Heat-Flux | -0.63 (0.74) | -2.13 (-2.94; 1.67) | 0.87 (0.41; 1.67) | 0.6 | 0.52 (0.47; 0.57) |

^a^Limits-of-agreement; ^b^Lin’s concordance correlation coefficient; ^c^Body mass index

**Online Resource 6** Mean bias over the course of measurement for a) Double-Sensor and b) Zero-Heat-Flux-Sensor


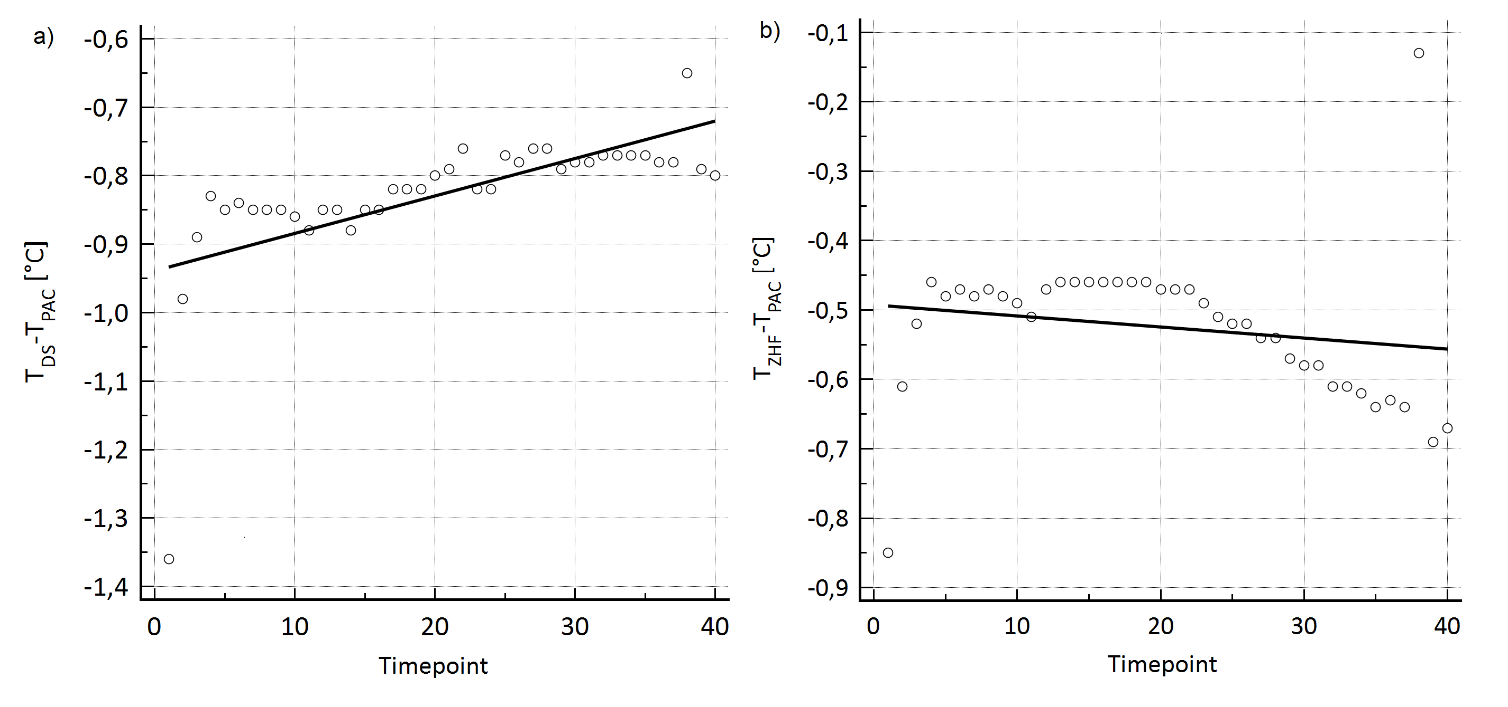


T_DS_: Double-Sensor temperature; T_ZHF_: Zero-Heat-Flux temperature; T_PAC_: Pulmonal artery temperature

**Online Resource 7** Receiver-operating-characteristic-curves for a) hyper- and b) hypothermia
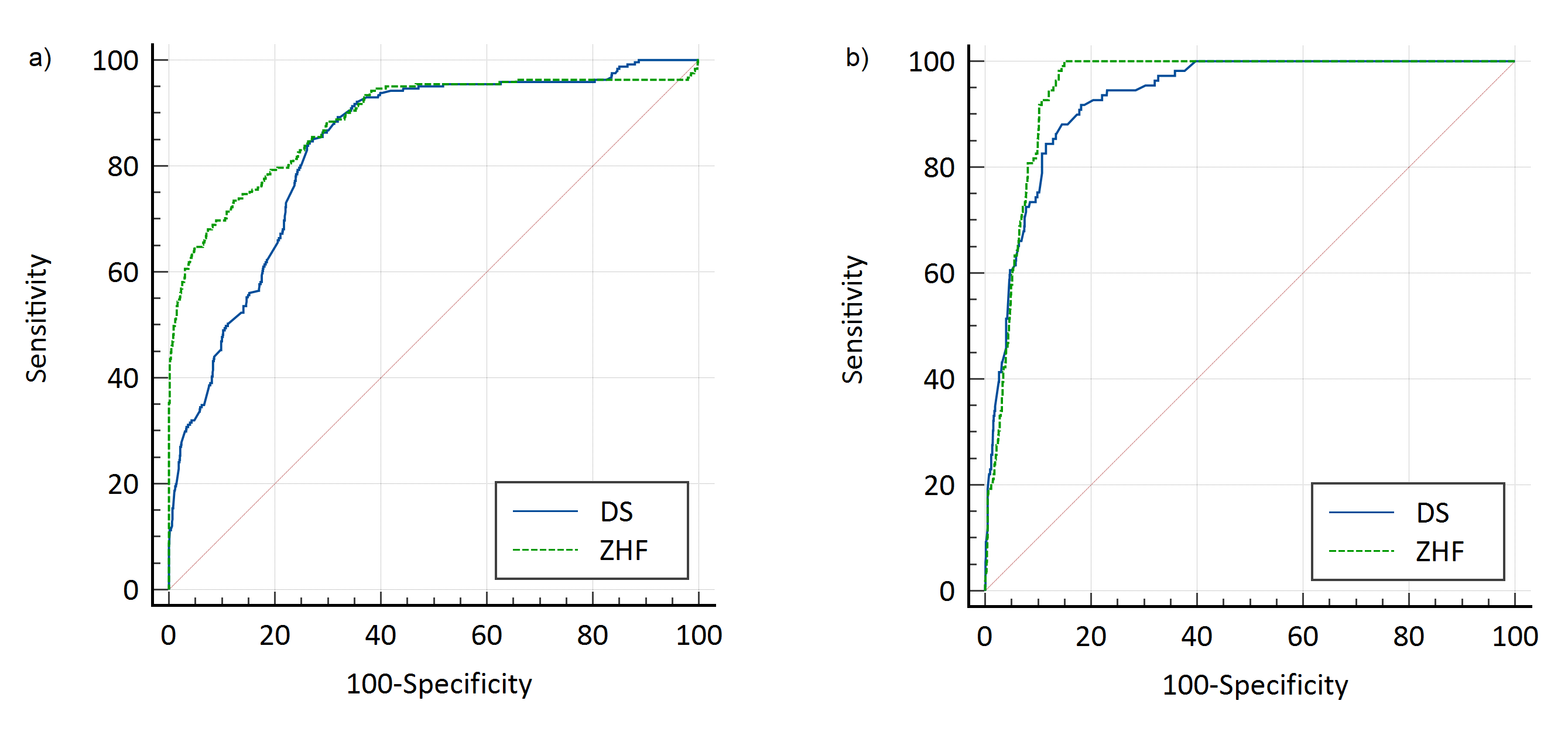


DS: Double-Sensor; ZHF: Zero-Heat-Flux-Sensor

**Online Resource 8** Mean temperature readings and mean bias per patient

| **ID** | **T_DS_ mean [°C]** | **T_ZHF_ mean [°C]** | **T_PAK_ mean [°C]** | **T_DS_-T_PAK_ mean [°C]** | **SD** | **T_ZHF_-T_PAK_ mean [°C]** | **SD** |
| --- | --- | --- | --- | --- | --- | --- | --- |
| 2 | 36.40 | 36.04 | 36.29 | 0.11 | 0.18 | -0.25 | 0.19 |
| 4 | 36.01 | 36.58 | 37.31 | -1.29 | 0.23 | -0.73 | 0.26 |
| 5 | 35.66 | 35.79 | 36.01 | -0.35 | 0.12 | -0.21 | 0.14 |
| 6 | 34.56 | 35.16 | 35.95 | -1.39 | 0.24 | -0.79 | 0.25 |
| 7 | 37.31 | 37.76 | 38.16 | -0.86 | 0.62 | -0.40 | 0.29 |
| 8 | 35.94 | 36.31 | 36.48 | -0.54 | 0.11 | -0.17 | 0.15 |
| 9 | 37.03 | 36.94 | 38.01 | -0.98 | 0.20 | -1.07 | 0.16 |
| 10 | 36.50 | 36.13 | 36.81 | -0.30 | 0.25 | -0.67 | 0.50 |
| 11 | 36.99 | 36.95 | 37.41 | -0.42 | 0.42 | -0.46 | 0.16 |
| 12 | 37.55 | 37.44 | 37.77 | -0.22 | 0.28 | -0.33 | 0.24 |
| 13 | 37.07 | 36.92 | 37.12 | -0.05 | 0.16 | -0.20 | 0.16 |
| 14 | 37.85 | 38.02 | 38.26 | -0.41 | 0.25 | -0.24 | 0.20 |
| 15 | 36.88 | 37.17 | 37.26 | -0.38 | 0.27 | -0.09 | 0.15 |
| 16 | 37.12 | 37.44 | 38.07 | -0.95 | 0.37 | -0.63 | 0.70 |
| 17 | 35.44 | 34.98 | 36.62 | -1.18 | 0.22 | **-1.64** | 0.24 |
| 18 | 37.02 | 37.66 | 37.83 | -0.81 | 0.17 | -0.17 | 0.13 |
| 19 | 37.01 | 36.56 | 37.63 | -0.61 | 0.28 | -1.06 | 0.29 |
| 20 | 35.79 | 36.72 | 37.35 | **-1.56** | 0.26 | -0.63 | 0.23 |
| 21 | 36.07 | 36.03 | 36.38 | -0.31 | 0.27 | -0.36 | 0.17 |
| 22 | 35.77 | 36.08 | 36.67 | -0.90 | 0.09 | -0.59 | 0.09 |
| 23 | 34.48 | 35.96 | 36.60 | **-2.12** | 0.32 | -0.64 | 0.20 |
| 24 | 37.19 | 37.22 | 37.48 | -0.28 | 0.21 | -0.26 | 0.24 |
| 25 | 36.50 | 36.77 | 37.90 | -1.40 | 0.32 | -1.12 | 0.18 |
| 26 | 37.65 | 37.70 | 37.55 | 0.11 | 0.17 | 0.15 | 0.11 |
| 27 | 36.54 | 37.52 | 37.59 | -1.05 | 0.12 | -0.08 | 0.12 |
| 28 | 35.92 | 36.64 | 36.72 | -0.80 | 0.09 | -0.08 | 0.12 |
| 29 | 37.03 | 37.19 | 37.49 | -0.45 | 0.17 | -0.29 | 0.11 |
| 30 | 36.07 | 36.56 | 37.09 | -1.01 | 0.30 | -0.53 | 0.35 |
| 31 | 36.74 | 37.50 | 37.97 | -1.23 | 0.18 | -0.46 | 0.19 |
| 32 | 36.67 | 36.77 | 36.98 | -0.30 | 0.12 | -0.21 | 0.09 |
| 33 | 35.63 | 36.17 | 36.71 | -1.08 | 0.15 | -0.54 | 0.20 |
| 34 | 35.02 | 34.73 | 36.99 | **-1.97** | 0.57 | **-2.26** | 1.07 |
| 35 | 35.86 | 36.37 | 37.07 | -1.21 | 0.21 | -0.71 | 0.19 |
| 37 | 36.14 | 36.91 | 37.05 | -0.91 | 0.14 | -0.13 | 0.14 |
| 39 | 36.31 | 36.84 | 37.25 | -0.94 | 0.22 | -0.40 | 0.14 |
| 40 | 37.37 | 37.96 | 38.54 | -1.18 | 0.93 | -0.58 | 0.25 |
| 42 | 34.96 | 35.56 | 35.67 | -0.71 | 0.12 | -0.11 | 0.14 |
| 43 | 37.23 | 37.09 | 37.33 | -0.09 | 0.11 | -0.23 | 0.10 |
| 44 | 34.75 | 35.21 | 37.13 | **-2.39** | 0.35 | **-1.92** | 0.59 |
| 46 | 37.41 | 37.62 | 38.00 | -0.59 | 0.20 | -0.38 | 0.18 |

ID: Patient-ID; T_DS_: Double-Sensor temperature; T_ZHF_: Zero-Heat-Flux temperature; T_PAC_: Pulmonal artery temperature
